# *Metarhiziumpuerense* (Hypocreales, Clavicipitaceae): a new species from Yunnan, south-western China

**DOI:** 10.3897/BDJ.12.e129087

**Published:** 2024-08-26

**Authors:** Jin Mei Ma, Zhi Qin Wang, Zhi Li Yang, Yue Chen, Song Yu Li, Hong Yu

**Affiliations:** 1 School of Ecology and Environment, Yunnan University, Kunming, China School of Ecology and Environment, Yunnan University Kunming China

**Keywords:** *Metarhizium*, morphology, phylogenetic analyses

## Abstract

**Background:**

As a genus within the Clavicipitaceae, *Metarhizium* exhibits rich morphological and ecological diversity, with a wide distribution and a variety of hosts. Currently, sixty-eight species of *Metarhizium* have been described.

**New information:**

A new species of *Metarhizium*, *M.puerense* (Hong Yu bis), was described in Pu'er City, Yunnan Province, south-western China. Based on morphological characteristics and multilocus phylogenetic analyses, *Metarhiziumpuerense* was confirmed to be phylogenetically related to *M.album*, but was clearly separated and formed a distinct branch. In contrast, the host of *Metarhiziumalbum* was plants and leafhoppers and that lepidopteran larvae were the host of *M.puerense*. The diagnostic features of *M.puerense* were solitary to multiple stromata and smooth-walled, cylindrical with rounded apices conidia.

## Introduction

*Metarhizium*, as a group with rich morphological and ecological diversity in Clavicipitaceae, is very rich in widely distributed and complex habitats (Bischoff et al. 2009). The type species *Metarhiziumanisopliae* (Metschn 1879) was used by Sorokīn (1883) to establish the new asexual genus *Metarhizium* (Tulloch 1976). Advances in molecular systematics have led to the emergence of multigene systematic analysis as a new technical method for the taxonomic identification of *Metarhizium* sp., combining functional protein genes with rDNA gene fragments (Sung et al. 2007and Kepler et al. 2012 and Kepler et al. 2014). In their study of genetic diversity within *Metarhizium* species, Driver et al. (2000) were the first to utilise molecular biology techniques. They solved the problem of classification at the species and varietal levels by identifying four variants in the *Metarhiziumanisopliae* complex groups, five variants in the *M.flavoviride* (Gams 1973) complex groups and delineating *M.album* (Petch 1931). In a multi-gene phylogenetic study of *Metarhiziumanisopliae* and *M.flavoviride* lineages, Bischoff et al. 2006 and Bischoff et al. 2009 elevated and accepted *Metarhizium* varieties to species rank by using additional protein-coding genes (*EF-1α*, *RPB1*, *RPB2* and TUB). The re-examination of *Metarhizium* and related genera led to the establishment of six new genera: *Keithomyces*, *Marquandomyces*, *Papiliomyces*, *Purpureomyces*, *Sungia* and *Yosiokobayasia* (*Mongkolsamrit et al. 2020*). *Chamaeleomyces* (Samson 1974) and *Nomuraea* spp. (Samson 1974), excluding *N.atypicola* (Samson 1974) and *Paecilomycesviridis* (Segretain 1964), were transferred to *Metarhizium*, and 19 new species of *Metarhizium* were reported. Other new species have been reported by Chen et al. 2018a and Chen et al. 2018b and Chen et al. 2018c and Chen et al. 2023 and Li et al. (2023). Currently, sixty-eight species of *Metarhizium* have been described.

According to the latest classification system, the *Metarhizium* genus belongs to the Fungi, Ascomycota, Sordariomycetes, Hypocreales and Clavicipitaceae. Its typical morphological characteristics are: Sexual form: Stromata single or multiple, unbranched or irregularly branched, mostly fleshy, with the main colours being pale yellow, green to greenish-brown or dark purple; fertile parts columnar or rod-shaped; perithecia partially or completely immersed; asci mainly columnar, ascospores linear, fusiform, breaking into secondary ascospores upon maturity or not. Asexual form: Growing rapidly on PDA medium, the colonies are flat and velvety, initially white and turn yellow-green or green after sporulation; phialides are morphologically diverse, single on the aerial hyphae or verticillate on the conidiophores; conidia are smooth, oval to columnar, spherical to subspherical, ovoid, aggregated in chains or clusters (Liang 2007). The typical characteristics of *M.puerense* were solitary to multiple stromata and smooth-walled, brownish in colour and producing a large number of green powdery conidia at the tip.

*Metarhizium* species that parasitise lepidopteran larvae were collected from Yunnan for this investigation. Phylogenetic location was elucidated, based on Bayesian Inference (BI) and Maximum Likelihood (ML) analyses, which involved concatenating sequences of the six loci. The results revealed that the species in question belong to the genus *Metarhizium*, specifically *Metarhiziumpuerense*.

## Materials and methods

**Collection and isolation of strains**

Specimens were collected from the broad-leaved evergreen forest of Pu'er, Yunnan Province, China, 2 August 2023, 22°71.33'N, 100°95.57'E, alt. 1358 m. The samples were preserved in sterile tubes and stored at 4°C. To obtain pure cultures, fresh specimens were cleaned and surface-sterilised by soaking in 30% hydrogen peroxide for approximately one minute. The samples were then washed with sterile water to remove residual hydrogen peroxide and the residual water was aspirated with a sterile filter paper. The worms were dissected on an ultra-clean bench, picked up with a sterilised scalpel with an appropriate amount of white tissue in the sclerotium centre, inoculated on potato dextrose agar medium (PDA: fresh potato 200 g/l, dextrose 20 g/l and agar 18 g/l) (Wang et al. 2020) and allowed to incubate at room temperature. The collected specimens were placed in the Yunnan Herbarium of the Yunnan University (YHH). The obtained strains were preserved at the Yunnan Fungal Culture Conservation Center (YFCC).

**Morphological characterization**

Fresh specimens, including the stromata and hosts, were photographed using a Canon 750D camera. For descriptions of colony appearance and microscopic features, the colonies on PDA plates were cultured for two weeks and the colony characteristics (size, texture and colour) were photographed with a Canon 700D camera to characterise the morphology of the colonies. Observations, measurements and photographs of the phialides and conidia were obtained using a light microscope (Olympus BX53).

**DNA extraction, PCR and sequencing**

DNA extraction was performed using a ZR Fungal DNA kit (Zymo, California, USA). DNA was preserved at -20˚C and used as a template for PCR amplification of the six loci. To amplify the largest and second-largest subunit sequences of RNA polymerase II (*RPB1* and *RPB2*), the primer pair RPB1-5'F and RPB1-5'R, as well as the primer pair RPB2-5'F and RPB2-5'R, were applied (Bischoff et al. 2006). The nuclear ribosomal small and large subunits (SSU and LSU) were amplified using the primer pairs used by 18S-CoF and 18S-CoR (Wang et al. 2015), as well as LR5 and LR0R (Vilgalys and Hester 1990 and Rehner and Samuels 1994). The translation elongation factor 1α (*EF-1α*) gene was amplified using the primer pair *EF1α-EF* and *EF1α-ER* (Bischoff et al. 2006 and Sung et al. 2007). PCR primers used to amplify the internal transcribed spacers were ITS4 and ITS5 (White et al. 1990). All PCR reactions were performed in a final volume of 50 μl and contained 25 μl of 2 × Taq PCR Master Mix (Tiangen, Beijing, China), 0.5 µl forward and reverse primers (10 μM), 1 μl template DNA (1 ng/μl) and 23 μl sterile distilled water. The polymerase chain reaction (PCR) was performed as described by Wang et al. (2015).

**Phylogenetic analysis**

The data matrix included 72 sequences from 48 species in *Metarhizium* and two out-group taxa. Sequences of six loci (ITS, SSU, LSU, *EF-1α*, *RPB1* and *RPB2*) were retrieved from GenBank. Sequences were aligned using MUSCLE software (Tamura et al. 2013). After alignment, the gene sequences were concatenated. *Clonostachysrosea* (GJS 90-227) and *Hydropisphaerapeziza* (CBS 102038) were designated as the outgroup taxa. Phylogenetic analyses were conducted using BI and ML methods with MrBayes v.3.1.2 and RaxML 7.0.3, respectively (Ronquist and Huelsenbeck 2003 and Stamatakis et al. 2008). The GTR+G+I model was determined using jModelTest version 2.1.4 (Darriba et al. 2012) with five million generations for the BI analysis. GTR+I was selected as the optimal model for the ML analysis and 1,000 rapid bootstrap replicates were performed on the dataset.

## Taxon treatments

### Metarhizium puerense

Hong Yu bis, J. M. Ma & Z.Q. Wang sp. nov.

5C2DB950-B9E9-513C-8098-724B940B0473

852903

#### Materials

- **Type status:** Holotype. **Occurrence:** occurrenceID: 5C808899-7FA6-5C36-8FA2-DBD6D69DBD82; **Taxon:** scientificName: Metarhiziumpuerense sp. nov.; **Location:** country: China; stateProvince: Yunnan; locality: Pu'er City, Simao District; verbatimElevation: 1358 m; verbatimLatitude: 22°71.33'N; verbatimLongitude: 100°95.57'E; **Identification:** identifiedBy: Hong Yu bis; **Event:** year: 2023; month: August; day: 2; **Record Level:** institutionID: YHH MP2308031; collectionID: YFCCMP 9458
- **Type status:** Other material. **Occurrence:** occurrenceID: E7C934B3-11EC-5ED2-A85F-CF9975A5C5E4; **Taxon:** scientificName: Metarhiziumpuerense sp. nov.; **Location:** country: China; stateProvince: Yunnan; locality: Pu'er City, Simao District; verbatimElevation: 1358 m; verbatimLatitude: 22°71.33'N; verbatimLongitude: 100°95.57'E; **Identification:** identifiedBy: Hong Yu bis; **Event:** year: 2023; month: August; day: 2; **Record Level:** institutionID: YHHMP 2308032; collectionID: YFCCMP 9459
- **Type status:** Other material. **Occurrence:** occurrenceID: D72AACD2-AF02-550B-8D65-667FC7E27EB7; **Taxon:** scientificName: Metarhiziumpuerense sp. nov.; **Location:** country: China; stateProvince: Yunnan; locality: Pu'er City, Simao District; verbatimElevation: 1359 m; verbatimLatitude: 22°71.33'N; verbatimLongitude: 100°95.57'E; **Identification:** identifiedBy: Hong Yu bis; **Event:** year: 2023; month: August; day: 2; **Record Level:** institutionID: YHHMP 2308033

#### Description

**Sexual morph**: Sexual morphs were not found.

**Figure 1.**
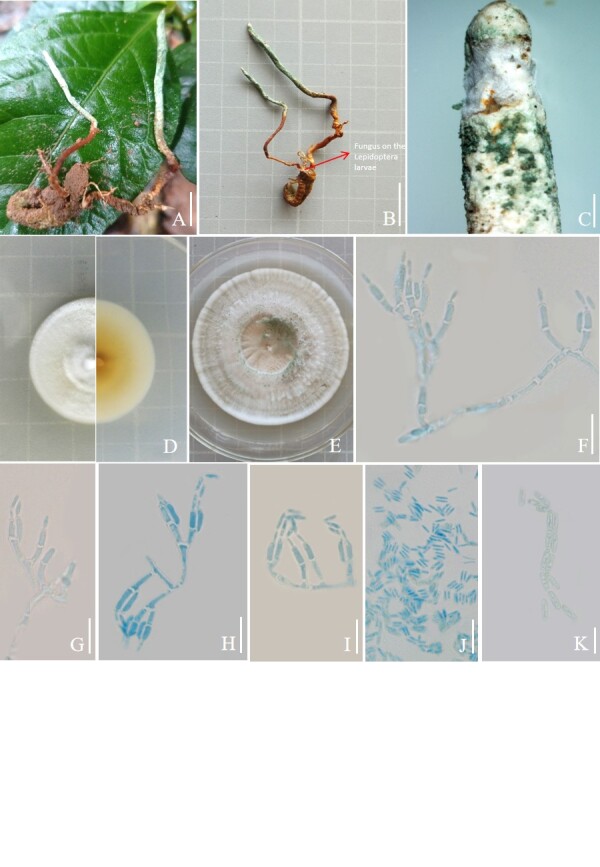
**Figure 1**. *Metarhiziumpuerense* (YFCCMP 9458). **A.** Stromata arising from hosts buried in soil. **B.** Fungus on the larvae of Lepidoptera. **C.** Apical part of stromata **D-E.** Culture characters on PDA (**D** = after 14 days, **E** = after 30 days). **F-I.** Conidiophores, phialides and conidia. **J-K.** Conidia. Scale bars: **A-E** = 1 cm. *F-J* = 10 µm. **K** = 5 µm.

**Asexual morph**: Stroma arising from the larvae of Lepidoptera larva buried in soil, solitary or multiple, brownish in colour and producing a large number of green powdery conidia at the tip. Colonies on PDA grew at 25°C, reaching 25-28 mm diam. in 14 days, cottony with high mycelium density, white to light yellow and reverse yellow; 45-52 mm in diameter in 30 days at 25°C, first white turning to green, powdery while sporulating, white mycelium at the margin. Hyphae septate, smooth-walled. Conidiophores smooth, cylindrical and erect. Phialides cylindrical, borne singly on aerial mycelium or whorled on conidial peduncle, 6.1-17.6 × 1.5-2.9 µm. Conidia were smooth-walled, ellipsoid to columnar, rounded at the tip, aggregated into chains or clusters, 3.8-7.1 × 1.3-2.1 µm (Fig. 1).

**Notes**: Phylogenetically, *Metarhiziumpuerense* is closely related to *M.album*, but differs in morphological characteristics. The morphological characteristics of *M.puerense* are as follows: stroma arising from the larva of Lepidoptera buried in soil, solitary or multiple, brownish in colour and producing a large number of green powdery conidia at the tip. *Metarhiziumalbum* was collected from plants and leafhoppers (Homoptera, Auchenorrhyncha) from rice. Moreover, *M.puerense* was indicated by its conidia size (3.8-7.1 × 1.3-2.1 µm), which was smaller than that of *M.album* (5-8 × 2-2.5 µm). The phialides of *M.puerense* (6.1-17.6 × 1.5-2.9 µm) was more slender than *M.album* (10-12.5 × 2-3.5 µm) (Michiel et al. 1987). Morphological comparisons of Metarhiziumpuerense with its related species (Table 2).

#### Etymology

Named after Pu'er City, where the species were first collected.

## Analysis

**Figure 2.**
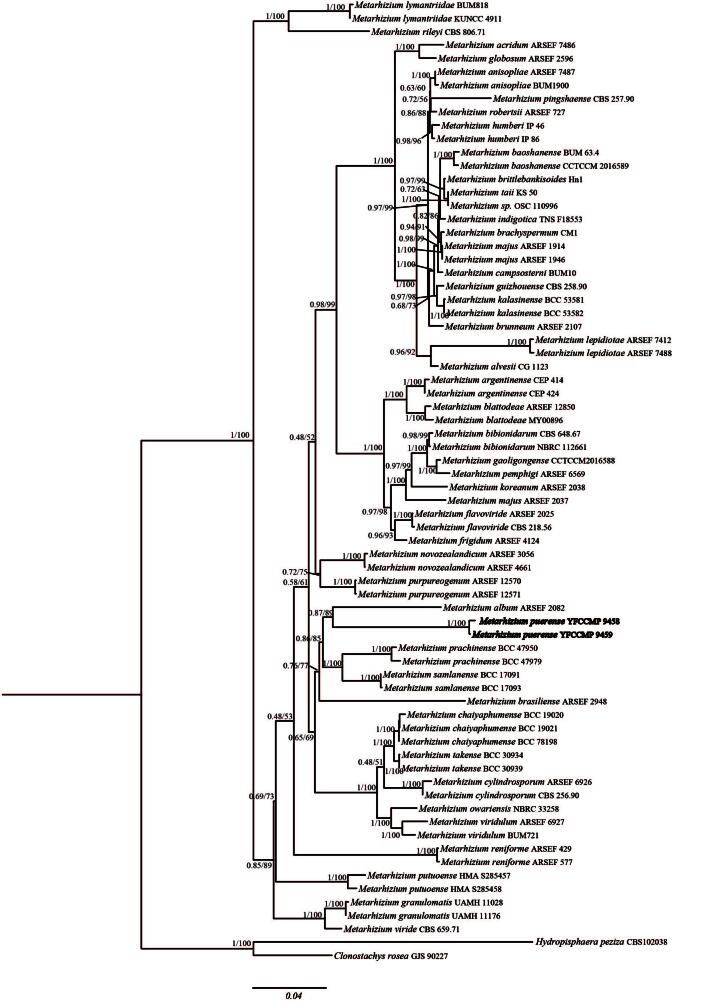
**Figure 2**. Phylogenetic placement of *M.puerense* was inferred from Maximum Likelihood (ML) and Bayesian Inference (BI) analyses, based on six loci (ITS, SSU, LSU, *EF-1α*, *RPB1* and *RPB2*).

These 49 taxa were used for phylogenetic analyses (Table 1). The combined six-locus dataset contained 4862 base pairs (bp) of sequences after alignment: 607 bp for ITS, 914 bp for SSU, 802 bp for LSU, 902 bp for *EF*-*1α*, 688 bp for *RPB1* and 1101 bp for *RPB2*. *Clonostachysrosea* (GJS 90227) and *Hydropisphaerapeziza* (CBS 102038) were designated as outgroup taxa in the phylogenetic tree. In phylogenetic trees based on both Bayesian Inference (BI) and Maximum Likelihood (ML) analyses, the samples collected in Yunnan formed a strongly-supported clade that was sister to *M.album* (BI posterior probability = 1.00, ML bootstrap =100%). This result indicates that it is a new species of *Metarhizium*, named *M.puerense* (Fig. 2).

## Discussion

To date, multi-locus phylogenetics, based on the joint analysis of ribosomal DNA and functional protein-coding genes, have been widely used in the phylogenetic study of fungi and have achieved many results (Sung et al. 2007 and Luangsa-ard et al. 2017 and Mongkolsamrit et al. 2020). In this study, we conducted an investigation, searched for and retrieved the *Metarhizium* nuclear gene sequences from the NCBI database. Subsequently, the sequences were compared with the obtained data. Additionally, a phylogenetic tree was constructed, based on multilocus database analyses (ITS, SSU, LSU, *EF-1α*, *RPB1* and *RPB2*) to elucidate the phylogenetic position of *M.puerense*. Phylogenetically, *Metarhiziumpuerense* is closely related to *M.album*. However, in terms of morphological characteristics, *M.puerense* parasitises the larvae of Lepidoptera, either solitary or multiple and produces a large number of green conidia. There were also differences in the sizes of phialides and conidia.

In the forests of Pu'er City, Yunnan Province, China, which are characterised by a warm and humid climate, a diverse array of entomopathogenic fungi thrive. Amongst these, *Metarhizium* is a fungal insecticide with large-scale production capabilities. It offers significant value owing to its environment-friendly nature, extended efficacy period and low resistance potential. This makes it an important asset for pest control. Hence, it is crucial to accurately identify the *Metarhizium* species and determine their host range to facilitate the development and utilisation of this potent insecticidal agent. In the current study, a new species collected from Pu'er City, *Metarhiziumpuerense*, is described. The phylogenetic and morphological evidence presented in this study supports the classification of the species as a new taxon within the genus *Metarhizium*. This research contributes to the expansion of diversity within *Metarhizium* species, enhances our understanding of host interactions, morphology, distribution and pure culture characteristics and provides valuable taxonomic and phylogenetic information for further detailed investigations of the genus. Additionally, this opens up new possibilities for the development of fungal insecticides.

In the investigation of entomogenous fungi resources in Yunnan, a new species of *Metarhizium* was discovered and identified. This work not only increases the diversity of species in the genus *Metarhizium*, enriches the biological fungal species resource pool in Yunnan Province, but also lays a certain foundation for the distribution of *Metarhizium* species in Yunnan Province and other regions in China. Additionally, it deepened our understanding of the morphology, distribution and pure culture characteristics of the *Metarhizium* genus and provided taxonomic and phylogenetic information for a more detailed study of the genus's systematics.

## Supplementary Material

XML Treatment for Metarhizium puerense

## Figures and Tables

**Table 1.** GenBank accession numbers of materials used in this study.

*Metarhiziumacridum*	ARSEF 7486	Orthoptera	HQ331458			EU248845	EU248897	EU248925
*Metarhiziumalbum*	ARSEF 2082	Hemiptera	AY375446	DQ522560	DQ518775	DQ522352	KJ398617	KJ398715
*Metarhiziumalvesii*	CG 1123	Soil				KY007614	KY007612	KY007613
*Metarhiziumanisopliae*	ARSEF 7487	Orthoptera	HQ331446			DQ463996	DQ468355	DQ468370
*Metarhiziumanisopliae*	BUM 1900	Soil	MH143803	MH143837	MH143820	MH143854	MH143869	MH143884
*Metarhiziumargentinense*	CEP 414	Blattodea: Blaberidae (*Epilampra* sp.)	MF784813			MF966620	MF966621	MF966622
*Metarhiziumargentinense*	CEP 424	Blattodea: Blaberidae (*Epilampra* sp.)				MF966624	MF966625	MF966626
*Metarhiziumbaoshanense*	BUM 63.4	Soil	KY264173	KY264178	KY264175	KY264170	KY264181	KY264184
*Metarhiziumbaoshanense*	CCTCCM2016589	Soil	KY264172	KY264177	KY264174	KY264169	KY264180	KY264183
*Metarhiziumbibionidarum*	CBS 648.67	Coleoptera: Scarabaeidae (*Cetoniaaurata*)				LC126075	LC125907	LC125923
*Metarhiziumbibionidarum*	NBRC 112661	Diptera (March fly larva)				LC126076	LC125908	LC125924
*Metarhiziumblattodeae*	ARSEF 12850	Blattodea: Ectobiidae	KU182915			KU182917	KU182918	KU182916
*Metarhiziumblattodeae*	MY00896	Blattodea	HQ165697	HQ165657	HQ165719	HQ165678	HQ165739	HQ16563
*Metarhiziumbrachyspermum*	CM1	Coleoptera	LC469747		LC469749	LC469751		
*Metarhiziumbrasiliense*	ARSEF 2948	Hemiptera	AF139854			KJ398809	KJ398620	KJ398718
*Metarhiziumbrittlebankisoides*	Hn1	Coleoptera				AB778556	AB778555	AB778554
*Metarhiziumbrunneum*	ARSEF 2107T	Coleoptera	KC178691		MH868397	EU248855	EU248907	EU248935
*Metarhiziumcampsosterni*	BUM 10	Soil	MH143798	MH143832	MH143815	MH143849	MH143864	MH143879
*Metarhiziumchaiyaphumense*	BCC 19020	Hemiptera: Cicadidae (*Cicada adult*)	HQ165695	HQ165654	HQ165716	HQ165675	HQ165737	HQ165635
*Metarhiziumchaiyaphumense*	BCC 19021	Hemiptera: Cicadidae (*Cicada nymph*)	HQ165696	HQ165655	HQ165717	HQ165676	HQ165738	HQ165636
*Metarhiziumchaiyaphumense*	BCC 78198	Hemiptera: Cicadidae (*Cicada nymph*)		KX369596	KX369593	KX369592	KX369594	KX369595
*Metarhiziumcylindrosporum*	ARSEF 6926	Hemiptera				KJ398814	KJ398625	KJ398723
*Metarhiziumcylindrosporum*	CBS 256.90	Hemiptera	MH862209		MH873892	KJ398783	KJ398594	KJ398691
*Metarhiziumflavoviride*	ARSEF 2025	Soil	AF138269			KJ398804	KJ398614	KJ398712
*Metarhiziumflavoviride*	CBS 218.56	Coleoptera	MH857590		MH869139	KJ398787	KJ398598	KJ398694
*Metarhiziumfrigidum*	ARSEF 4124	Coleoptera	HM055448			DQ464002	DQ468361	DQ468376
*Metarhiziumgaoligongense*	CCTCCM2016588	Soil	KY087808	KY087812	KY087816	KY087820	KY087824	KY087826
*Metarhiziumglobosum*	ARSEF 2596	Lepidoptera	HQ331459			EU248846	EU248898	EU248926
*Metarhiziumgranulomatis*	UAMH 11028	*Chamaeleocalyptratus*	HM195305	HM635076	HM195304	KJ398781		KJ398688
*Metarhiziumgranulomatis*	UAMH 11176	*Chamaeleocalyptratus*	HM195306		HM635078	KJ398782	KJ398593	KJ398689
*Metarhiziumguizhouense*	CBS 258.90	Lepidoptera larva	MH862211		MH873894	EU248862	EU248914	EU248942
*Metarhiziumhumberi*	IP 46	Soil				MH837574	MH837556	MH837565
*Metarhiziumhumberi*	IP 86	Soil				MH837576	MH837558	MH837567
*Metarhiziumindigoticum*	TNS F18553	Lepidoptera larva	JN049874	JF415952	JF415968	JF416010	JN049886	JF415992
*Metarhiziumkalasinense*	BCC 53581	Coleoptera larva	KC011178	KC011174	KC011182	KC011188		
*Metarhiziumkalasinense*	BCC 53582	Coleoptera larva	KC011179	KC011175	KC011183	KC011189		
*Metarhiziumkoreanum*	ARSEF 2038	Hemiptera	HM055431			KJ398805	KJ398615	KJ398713
*Metarhiziumlepidiotae*	ARSEF 7412	Coleoptera	HQ331455			EU248864	EU248916	EU248944
*Metarhiziumlepidiotae*	ARSEF 7488	Coleoptera	HQ331456			EU248865	EU248917	EU248945
*Metarhiziumlymantriidae*	BUM 818		OM955147	OM951242	OM951247	OM988196	OM988192	OM988188
*Metarhiziumlymantriidae*	KUNCC 4991		OM955148	OM951243	OM951248	OM988197	OM988193	-
*Metarhiziummajus*	ARSEF 1914	Coleoptera	HQ331445			EU248868	EU248920	EU248948
*Metarhiziummajus*	ARSEF 1946	Coleoptera	HM055450			EU248867	EU248919	EU248947
*Metarhiziumminus*	ARSEF 2037	Hemiptera	AF138271	AF339580	AF339531	DQ522353	DQ522400	DQ522454
*Metarhiziumnovozealandicum*	ARSEF 3056	Soil				KJ398810	KJ398621	KJ398719
*Metarhiziumnovozealandicum*	ARSEF 4661	Soil				KJ398811	KJ398622	KJ398720
*Metarhiziumowariense*	NBRC 33258	Hemiptera	JN049883	HQ165669	HQ165730	JF416017	KJ398596	JF415996
*Metarhiziumpemphigi*	ARSEF 6569	Hemiptera: Apididae	-	-	-	KJ398813	KJ398624	KJ398722
*Metarhiziumpinghaense*	CBS 257.90	Coleoptera	HQ331450	-	MH873893	EU248850	EU248902	EU248930
*Metarhiziumprachinense*	BCC 47950	Lepidoptera	KC011176	KC011172	KC011180	KC011186	KC011184	-
*Metarhiziumprachinense*	BCC 47979	Lepidoptera	KC011177	KC011173	KC011181	KC011187	KC011185	-
*Metarhiziumpurpureogenum*	ARSEF 12570	Soil				LC126079	LC125911	LC125922
*Metarhiziumpurpureogenum*	ARSEF 12571	Soil			AB700552	LC126078	LC125913	LC125920
*Metarhiziumputuoense*	HMAS 285457	Coleoptera (larva)		OQ981977	OQ981970	OQ980403	OQ980411	
*Metarhiziumputuoense*	HMAS 285457	Coleoptera (larva)		OQ981978	OQ981971	OQ980404	OQ980412	
***Metarhiziumpuerense***	**YFCCMP 9458**	**Lepidoptera**	**PP733948**	**PP733950**	**PP733952**	**PP776150**	**PP776152**	**PP776154**
***Metarhiziumpuerense***	**YFCCMP 9459**	**Lepidoptera**	**PP733949**	**PP733951**	**PP733953**	**PP776151**	**PP776153**	**PP776155**
*Metarhiziumreniforme*	ARSEF 429	Orthoptera	DQ069284	HQ165671	HQ165733	HQ165690		HQ165650
*Metarhiziumreniforme*	ARSEF 577	Orthoptera: Tettigoniidae	DQ069283	HQ165672	HQ165734	HQ165691		HQ165651
*Metarhiziumrile*yi	CBS 806.71	Lepidoptera: Noctuidae (*Trichoplusiani*)	AY624205	AY526491	MH872111	EF468787	EF468893	EF468937
*Metarhiziumrobertsii*	ARSEF 727	Orthoptera	HQ331453			DQ463994	DQ468353	DQ468368
*Metarhiziumsamlanense*	BCC 17091	Hemiptera: *Cicadellidae* (adult)	HQ165707	HQ165665	HQ165727	HQ165686		HQ165646
*Metarhiziumsamlanense*	BCC 17093	Hemiptera: *Cicadellidae* (adult)	HQ165709	HQ165666	HQ165728	HQ165687	HQ165746	HQ165647
*Metarhiziumtakense*	BCC 30934	Hemiptera: Cicadidae (*nymph*)	HQ165698	HQ165658	HQ165720	HQ165679	HQ165740	HQ165639
*Metarhiziumtakense*	BCC 30939	Hemiptera: Cicadidae (*nymph*)	HQ165699	HQ165659	HQ165721	HQ165680	HQ165741	HQ165640
*Metarhiziumviride*	CBS 659.71	Hemiptera: Cicadidae (*nymph*)	HQ165714	HQ165673	HQ165735	HQ165692		HQ165652
*Metarhiziumviridulum*	ARSEF 6927	Chamaeleo lateralis				KJ398815	KJ398626	KJ398724
*Metarhiziumviridulum*	BUM 721	Hemiptera	MH143808	MH143842	MH143825	MH143859	MH143874	MH143889
*Metarhiziumtaii*	KS 50	Soil		GU979940-	GU979949	GU979958		GU979972
*Metarhizium* sp.	OSC 110996			EF468974	EF468832	EF468773	EF468880	EF468928
*Clonostachysrosea*	GJS 90-227			AY489684	AY489716	AY489611		
*Hydropisphaerapeziza*	CBS 102038			AY489698	AY489730	AY489625	AY489661	DQ522444

**Table 2.** Morphological comparisons of *Metarhiziumpuerense* with its related species.

**Species**	**Host**	**Stromata**	**Fertile part**	**Colony on PDA**	**Anamorph**	**P hialides (µm)**	**Conidia (µm)**	**References**
***M* . *puerense***	Lepidoptera larva	Solitary or multiple, 2.6–4.7 cm long, 1.2– 1.5mm broad	Cylindrical to clavate, contains a large number of green conidia, 1–2 cm long, 1–1.5 mm broad	White dense mycelium, producing green spores later	Chain shape, clumping together	Solitary or in whorls of 2, 6.1–17.6 × 1.5–2.9	Ovoid to elliptical, 3.8–7.1 × 1.3–2.1	This study
***M.*** ***album***	Leafhoppers			Pure white to yellowish white, or greyish white becoming pinkish to fawn to pale brown upon sporulation	Conidial chains	Clavate phialides, solitary or in whorls of 2–5,10–12.5 × 2–3.5	Narrowly ellipsoid or ovoid, (3–)4–6× l.5–2.5	Michiel et al., 1987
***M.*** ***brasiliense***	Leafhoppers (Hemiptera: Cicadellidae)			White to cream, becoming dark green to bluish green			Short conidia, 5.5–9× 2.5–3.5	Kepler et al., 2014
***M.samlanense***	Leafhoppers (Hemiptera: Cicadellidae)			At first white turning green due to conidiation	Conidial chains	Phialides are short and cylindrical, 5–7 × 2–3	Green, globose,3 × 5	Jennifer Luangsa-ard et al., 2016
***M.prachinense***	Lepidoptera larva	Stromata usually branched, 50–86 × 1–2 mm, broad	Cylindrical with pointed ends, white, pale yellow to grayish yellow, 0.8–1.7 × 1 mm	Initially colorless, turning green due to the production of green conidia	Conidial chains	Ovoid to obpyriform with short distinct neck, 3–5×2	Subglobose, green, 3–5 × 1.5–2.5	Jennifer Luangsa-ard et al., 2016
